# Mapping the research, policy, and clinical management landscape of post-tuberculosis lung disease: A scoping review

**DOI:** 10.1371/journal.pgph.0007150

**Published:** 2026-08-28

**Authors:** Yochanan Hoppenstein, Olena Ivanova, Salome Charalambous

**Affiliations:** 1 School of Public Health, University of the Witwatersrand, Johannesburg, South Africa; 2 Institute of Infectious Diseases and Tropical Medicine, LMU University Hospital, LMU Munich, Munich, Germany; 3 SPHERE CRE, Department of General Practice, School of Public Health and Preventive Medicine, Monash University, Melbourne, Victoria, Australia; 4 The Aurum Institute, Johannesburg, South Africa; University of Sydney, AUSTRALIA

## Abstract

Tuberculosis (TB) can result in debilitating long-term health consequences, most notably post-tuberculosis lung disease (PTLD). However, these conditions remain insufficiently integrated into global policy and routine care, contributing to fragmented care for millions of TB survivors. This scoping review maps the current landscape to identify critical gaps and support the harmonisation of research, policy, and practice. We conducted a scoping review of peer-reviewed research and global guidance documents published between June 2017 and May 2025. The search for peer-reviewed research focused specifically on PTLD management, whereas the search for guidance documents used broader terms for post-tuberculosis (post-TB) sequelae to assess how PTLD is conceptualised within the wider policy context. Data on recognition, management recommendations, and classification were systematically extracted and thematically synthesised. We included 28 documents: 14 research publications and 14 global guidance documents. The research literature offered detailed management content, including diagnostic assessment, pulmonary rehabilitation and chronic care approaches, acute-on-chronic complication management, and follow-up schedules. In contrast, only 8 of 14 guidance documents provided management recommendations relevant to PTLD. Recurring guidance recommendations included structured post-TB respiratory assessment, multidisciplinary care incorporating rehabilitation and psychosocial support, and clinical, radiological, and functional follow-up, whereas acute-on-chronic management was addressed far less consistently. Overall, we found a translation gap between evidence generation in research and its operationalisation in global guidance. Despite growing awareness and some recent policy progress, the global response to PTLD remains hampered by inconsistent and incompletely operationalised guidance, as well as the absence of standardised definitions, classification, and programmatic indicators. Accelerating the translation of research into integrated policy and practice is needed to improve the long-term health and quality of life of TB survivors.

## Introduction

Tuberculosis (TB) remains a major global health challenge, with over 155 million TB survivors worldwide, the majority living in low- and middle-income countries (LMICs) [1]. In 2023, 8.2 million new TB cases were diagnosed, the highest recorded since systematic monitoring began in the mid-1990s [2]. Yet, TB’s impact extends well beyond active infection; millions of survivors experience chronic, debilitating post-tuberculosis (post-TB) sequelae affecting multiple organ systems. These sequelae range from respiratory impairment to neurological, musculoskeletal, and cardiovascular dysfunction, and carry substantial socio-economic and public health consequences, including job loss, social exclusion, and stigma [3].

Among these sequelae, post-tuberculosis lung disease (PTLD) is notably prevalent, affecting an estimated 15–50% of TB survivors, with the highest rates in LMICs [4,5]. Factors associated with PTLD include delays in TB diagnosis and initiation of effective treatment, drug-resistant or recurrent TB, and smoke exposures [3,6–8]. Clinical patterns vary widely and include bronchiectasis, cavitation, pulmonary hypertension, obstructive lung disease, and aspergillus-related disease. These may be asymptomatic, present with chronic symptoms such as breathlessness and cough, or, in some cases, lead to severe acute-on-chronic exacerbations like haemoptysis [9]. Despite its substantial burden, PTLD remains poorly recognised within global health policies, in part because terminology and case definitions remain inconsistent, and global TB programme guidance and monitoring emphasise microbiological outcomes and survival during treatment, with limited focus on post-treatment morbidity and follow-up [4,6,10,11]. There are currently no internationally agreed consensus guidelines for its systematic detection, classification, or management [3].

A 2018 scoping review by Van Kampen et al. was the first to systematically assess international research publications and global guidance documents for recognition of TB sequelae, identifying only three international guidelines with any reference to TB sequelae, and none providing explicit detection criteria or management recommendations [12]. Since that review, the volume and scope of both peer-reviewed research and global guidance documents have expanded, with new studies probing pathophysiology, disease burden, and potential care strategies, and landmark reviews beginning to synthesise this evidence into preliminary recommendations. Yet the field remains fragmented: definitions and measurement of PTLD and related effects vary widely, and most recommendations have not been operationalised. In the absence of dedicated interventions, much current guidance is adapted from other chronic conditions and rests primarily on expert opinion [3,7,9]. Although several international guidance documents now acknowledge post-TB long-term effects, the depth and practical utility of this recognition have yet to be systematically examined.

Despite advances, the major organisations that monitor and advise on TB management such as the World Health Organization (WHO) and United States Agency for International Development (USAID) have yet to establish a clear and integrated response to PTLD, resulting in implementation gaps at national levels [13,14].

Accurately navigating the evolving research and guideline landscape is further complicated by variation in definitions and classifications across studies. Contested elements include when the post-TB period begins and whether definitions should include asymptomatic TB survivors with abnormal imaging [15]. The 2019 International PTLD Symposium called for standardised terminology, leading to the development of some consensus definitions [4]. However, adoption remains partial, with ongoing implications for comparability across burden estimates and management recommendations.

Overall, PTLD research and policy are evolving rapidly but remain fragmented across definitions, service integration, and operationalised care, limiting coordinated policy and clinical responses for TB survivors.

This scoping review serves as a navigational tool for research and policy. Unlike prior reviews, it systematically synthesises both peer-reviewed research and global guidance documents published since 2017, aiming not only to map how post-tuberculosis lung disease (PTLD) is recognised and managed, but also to clarify the key practical challenges to its integration in care systems. Because PTLD is variably recognised as a distinct entity or subsumed within broader post-TB sequelae terminology, this review considers both explicit PTLD references and broader post-TB sequelae framing in global guidance documents.

The aim of this review is to provide a practical resource for clinicians, policymakers, and researchers navigating PTLD clinical management across both the research literature and global guidance documents. To achieve this, we first map how recent research and global guidance recognise and manage PTLD, situating this within the broader context of how global guidance addresses other post-TB sequelae. Second, we compare the extent and depth of PTLD management across these evidence streams. Finally, we synthesise current recommendations and identify persistent gaps in definitions, classification, practical implementation, and integration into existing frameworks.

## Methods

### Study design

This scoping review was conducted in accordance with Arksey and O’Malley’s five-stage framework [16], which provides a structured yet flexible approach for topics with heterogeneous, evolving, and insufficiently consolidated evidence. Given our aim to map the breadth of evidence and identify policy and implementation gaps, rather than assess intervention effectiveness, a scoping review was chosen over a systematic review. Reporting was guided by the PRISMA Extension for Scoping Reviews [17]. No review protocol was registered or published. This review deliberately focused on secondary literature (research syntheses, clinical standards, and global guidance documents), excluding primary studies, to prioritise breadth, conceptual integration, and relevance for policy and guideline development.

### Search strategy

A comprehensive, two-pronged search strategy was implemented to identify relevant peer-reviewed research publications and global TB guidance documents.

For peer-reviewed publications, PubMed was selected as the primary database because of its extensive indexing of biomedical and TB-related research. Semantic Scholar was used as a supplementary search tool to identify potentially relevant records through its similar-paper recommendation features; these were manually screened against the same predefined eligibility criteria as PubMed records. The search was initially completed in April 2024 and covered publications between June 2017 and April 2024, building upon the 2018 review by Van Kampen et al. The search was re-run on 15 May 2025 to capture recent literature, serving as the final inclusion cut-off for both search streams. To accommodate evolving terminology and capture conceptual breadth, a wide range of search terms was used, including combinations of “post-tuberculosis lung disease”, “PTLD”, “TB complications”, “guidelines”, “interventions”, and “rehabilitation”, among others. In addition, studies that clearly referenced unrelated diseases (e.g., lymphoma, transplantation, Lyme disease) or which did not focus on post-tuberculosis lung disease or on its management were excluded through title and keyword filtering. The full search strategy, including PubMed search details, is provided in S1 Appendix. Screening and selection consisted of an initial screening of all titles and abstracts in line with the eligibility criteria. Full texts of potentially relevant articles were then reviewed in detail. Records with uncertain eligibility were independently assessed by two co-authors, and disagreements were resolved by consensus.

In parallel, global TB guidance documents were identified through targeted searches of major international health organisations, including the WHO, Stop TB Partnership, Global Fund, United Nations, Centers for Disease Control and Prevention (CDC), European Respiratory Society (ERS), USAID, and World Health Partners. Supplementary searches were conducted in the Guidelines International Network (GIN), TRIP Database, International Guideline Library, Cochrane Library, and PubMed. This process was iterative and included citation tracking. Where relevance was unclear from the title, summary, or stated scope, documents were searched in full text using predefined terms related to post-TB states and sequelae, post-treatment follow-up, disability, rehabilitation, palliation, complications, long-term outcomes, and chronic disease. Documents were not included or excluded solely on the basis of a keyword match; eligibility was determined using the criteria described below. The search was re-run on 15 May 2025 to capture recent literature, serving as the final inclusion cut-off for both search streams.

### Eligibility criteria

Eligibility criteria were defined in advance and applied systematically. Sources were included only if they were available in English and published or last updated between June 2017 and May 2025.

For research publications, inclusion was limited to those published in peer-reviewed journals. Only secondary literature, such as reviews, consensus statements, or expert syntheses, was included. Articles needed an explicit focus on post-tuberculosis lung disease and had to present practical clinical or programmatic management recommendations (e.g., clinical recommendations, expert opinion, or evidence synthesis). Eligible article types included reviews (whether clinical, scoping, systematic, or other), consensus statements, and peer-reviewed conference reports. Articles were excluded if they were primary research studies, focused exclusively on a single intervention or a narrow population subgroup (e.g., studies solely involving HIV-positive populations or targeted for a specific region), or were not peer-reviewed (such as editorials or news items). Additionally, research publications whose principal objective was centred on epidemiological burden, pathophysiology, aetiology, or risk factors were excluded.

**Fig 1:**
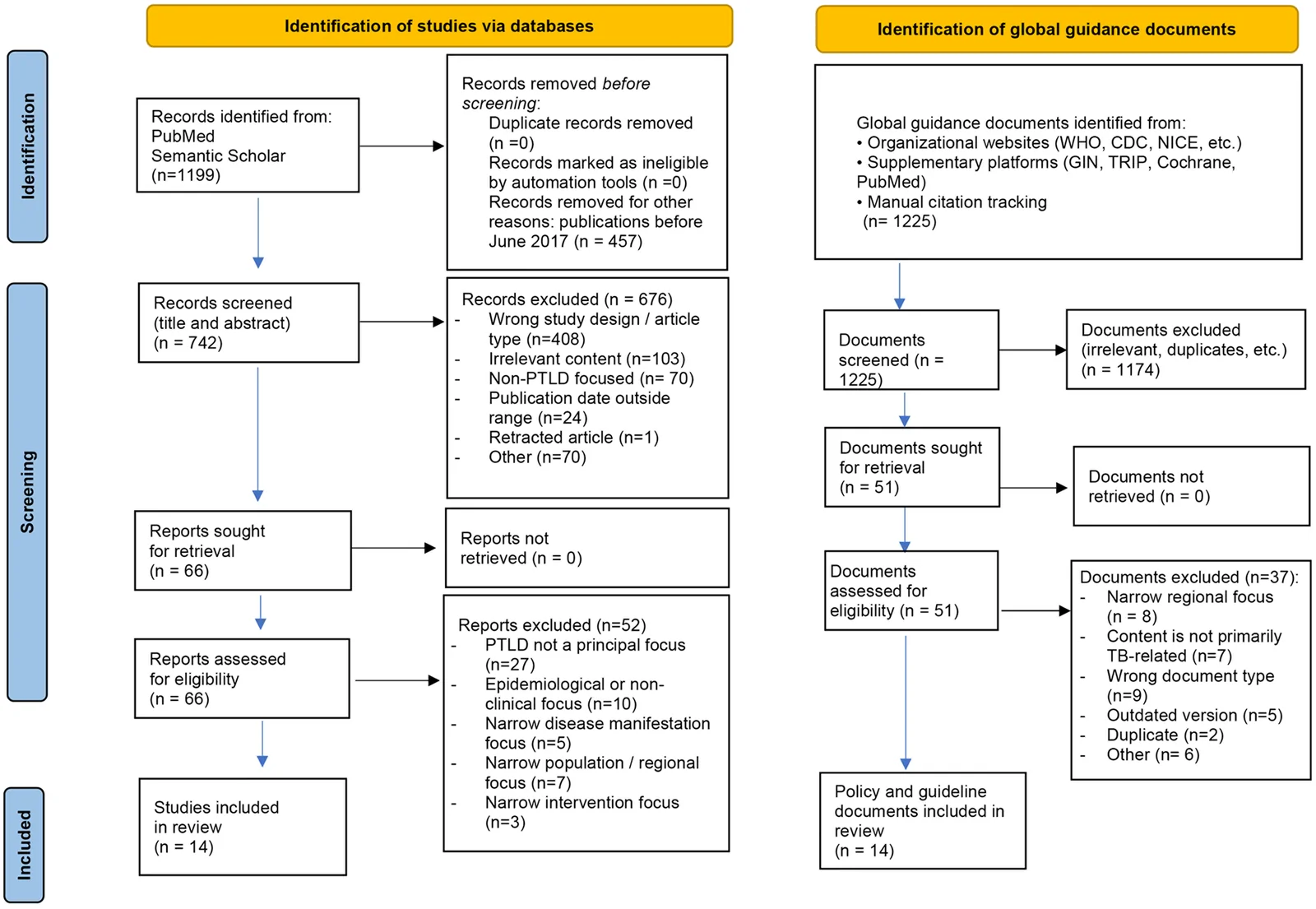
PRISMA-ScR flow diagram illustrating the identification, screening, eligibility, and inclusion of sources in the review. PRISMA 2020 format adapted to reflect the inclusion of both database-driven and targeted evidence streams.

For global guidance documents, inclusion criteria specified that documents had to be official guidelines, policy documents, consensus statements, implementation resources, monitoring reports, or strategic frameworks issued or formally endorsed by an international health authority or TB-focused organisation. Documents were eligible if they were global TB documents relevant to the broader TB care continuum, including post-treatment care or long-term outcomes after TB. Use of explicit PTLD or post-TB sequelae terminology was not required for inclusion in the global guidance stream. The results of the search and screening process are summarised in a PRISMA-style flowchart (see Fig 1).

National and sub-national policy documents were not systematically included, and materials published in languages other than English were excluded. Patient, survivor, and health worker perspectives were not directly represented in the included sources.

### Data extraction

Data extraction followed Arksey and O’Malley’s framework, with iterative refinement throughout. The process was initially conducted by the lead reviewer and subsequently cross-checked for consistency by two independent reviewers. Minor discrepancies were resolved through consensus, ensuring alignment with the study objectives and extraction framework.

Documents were annotated using Foxit PDF Editor Pro (v13.1 and later, 2024). Data extraction and charting were performed in Microsoft Excel (2024–2025). Reference management was handled in Zotero versions 6 and 7.

For each included document, the following variables were extracted: document details (including type, year, and source); verbatim terminology used for PTLD or post-TB sequelae, where present; predefined management domains addressed, including primary prevention, screening & diagnosis, chronic management, acute-on-chronic management, and long-term monitoring and follow-up; and any emergent themes, implementation considerations, and identified gaps. For global guidance documents, extraction also noted whether PTLD, other respiratory post-TB sequelae, non-respiratory post-TB sequelae, or broader TB-associated disability were explicitly recognised, and captured the content of management recommendations relevant to the predefined PTLD management domains.

Where guidance documents or research articles used non-standard or ambiguous terminology, or conflated domains (e.g., rehabilitation and palliation), notes were made to support later synthesis and ensure accurate thematic mapping. The full data extraction process is included in S1 Appendix.

### Management domains and coverage classification

Five PTLD management domains, representing the standard clinical continuum of care, were defined: primary prevention (strategies to mitigate lung injury before or during TB disease, prior to PTLD diagnosis); screening and diagnosis (processes aimed at identifying PTLD or those at risk, typically post-TB treatment); chronic management (ongoing interventions for established PTLD to improve function, prevent progression, and optimise quality of life); acute-on-chronic management (interventions addressing acute exacerbations or complications superimposed on chronic PTLD); and long-term monitoring and follow-up (ongoing assessment and review of PTLD patients over time to monitor progression, detect complications, and guide further care).

With respect to classifying the extent of coverage across the management domains, we applied a five-tier framework. *Extensive coverage* was assigned when a document included a dedicated section, figure, table, detailed discussion across multiple areas, or specific tools or protocols, any one of which was sufficient. *Superficial coverage* applied when the topic was explicitly discussed but only briefly and without a dedicated section or practical detail. *Brief mention* indicated that the topic was named but only in passing, with no elaboration or actionable content. *Acknowledged implicitly* was used when the topic was not explicitly named but could be reasonably inferred from broader language (e.g., “comprehensive care”), with justification documented. *Not addressed or unclearly referenced* was assigned when the topic was absent or too vague to interpret with confidence.

### Ethics and AI usage

As this study was a review of published literature and did not involve the collection of primary data or the participation of human subjects, ethical approval was not required.

During the preparation and revision of this manuscript, OpenAI’s GPT-4, GPT-4.1 and GPT-5.6 Sol, together with Anthropic’s Claude Opus 4.8, were used for language editing (clarity, grammar, and phrasing) in the Introduction, Methods, Results, and Discussion. All AI outputs were reviewed and approved by the authors. No AI tools were used for any aspect of data analysis or interpretation.

## Results

### Peer-reviewed research articles

#### Search outcomes.

A total of 742 unique records were screened for eligibility. Following title and abstract screening, 66 full-text articles were assessed, and 14 peer-reviewed research publications were included in the review (Fig 1; S1 Table). These publications were published between 2018 and 2025. Table 1 summarises their characteristics, including article type, population focus, geographic scope, and the general nature of PTLD content described by each source. The included publications comprised systematic/scoping reviews, clinical/narrative reviews, and consensus-based reports.

**Table 1:** Characteristics of included peer-reviewed secondary publications and orientating overview of PTLD focus.

Year	Author	Article type	Population focus (standardised)	Geographic scope	Nature of PTLD content (orientation only)
2018	Van Kampen [12]	Scoping review	TB survivors	International	Evidence and policy landscape mapping for PTLD; gaps in guidance and definitions noted
2020	Allwood et al. [4]	Conference report	TB survivors (adults and children)	Stellenbosch, South Africa (International Post-Tuberculosis Symposium)	Symposium report outlining PTLD definitions, multidisciplinary framing, and research/advocacy agenda
2020	Akkerman et al. [18]	Clinical review (narrative)	TB survivors with post-treatment sequelae and impaired lung function	Not specified	Long-term post-TB/PTLD management components (assessment, rehabilitation, nutrition, immune modulation)
2021	Allwood et al. [9]	Clinical review (narrative)	TB survivors (pulmonary TB; includes childhood TB discussion)	International / global	Staged clinical framework across the post-TB timeline and associated management approaches
2021	Meghji et al. [19]	Clinical review (narrative)	TB survivors	LMICs	PTLD within chronic respiratory diseases; health-system integration challenges
2021	Migliori et al. [7]	Clinical consensus-based standards	TB survivors (treatment completion)	Global	Consensus-based clinical standards; PTLD care including pulmonary rehabilitation assessment, long-term management, and follow-up
2022	Pontali et al. [6]	Scoping review	Post-treatment TB survivors and post-COVID survivors	International literature (PubMed search)	Post-TB vs post-COVID rehabilitation synthesis; comparative framework; pulmonary-rehabilitation component table
2022	Meca et al.[20]	Clinical review (narrative)	TB survivors with post-TB sequelae (post-treatment)	Not specified	Management algorithm linking pathogenic mechanisms to treatment strategies
2023	Nightingale et al.[3]	Clinical consensus-based review	TB survivors	Worldwide	Post-TB health review including respiratory and non-respiratory sequelae
2024	Sehgal et al. [8]	Clinical review (narrative)	TB survivors with PTLD-AFO	Global	PTLD-AFO classification and phenotype-tailored management guidance
2024	Alene et al. [11]	Systematic review and meta-analysis	Adults or children with TB	12 countries (included studies)	Preventive interventions for post-TB sequelae; strategies discussed include pulmonary rehabilitation
2024	Seo et al. [21]	Clinical review (narrative)	TB survivors with PTLD (post-treatment completion)	Not specified	Subtype-specific long-term PTLD care; practical flowchart
2024	Allwood et al. [15]	Conference report	TB survivors	Stellenbosch, South Africa (international symposium); delegates representing 31 countries	Stakeholder workshop report with Delphi process; definitions, research priorities, multidisciplinary considerations
2025	Meghji et al. [10]	Clinical review (narrative)	TB survivors (children, adolescents, and adults)	International	Overview structured around prevention, diagnosis, and care; implementation challenges and research priorities

Illustrative examples of management content across the five predefined PTLD management domains in the research literature are shown in Table 2.

**Table 2:** Illustrative management content in the research literature, by PTLD management domain.

Management domain	Illustrative management content
Primary prevention	Upstream prevention: social determinants, early diagnosis, novel treatment regimens, host-directed therapies [10] Prevention taxonomy: host- vs pathogen-directed targets; active TB disease vs post-treatment phase [15]
Screening and diagnosis	Modality appraisal for resource-constrained settings: symptom screening, spirometry, chest radiography, oxygen saturation, exercise testing [10] Essential investigations for suspected PTLD: clinical assessment, chest radiography, PFTs, 6MWT, QoL assessment [7] PTLD-AFO diagnostic algorithm: spirometry and CT first-line, phenotype-guided targeted workup [8] End-of-treatment lung function evaluation for impairment and rehabilitation eligibility [4] Post-cure baseline assessment: imaging and spirometry [21] Outcome-to-instrument mapping: SGRQ, EQ-5D [15]
Chronic management	Pulmonary rehabilitation: aerobic exercise, strength training, IMT, airway clearance, nutritional support; resource-limited adaptations [7] Pharmacological and procedural interventions for symptom control and structural complications [8,9,21] Tertiary prevention: patient education, counselling, vaccination, psychosocial support [8,9,21] Intervention framework: pathogen- vs host-directed target; during-TB vs post-treatment phase [15]
Acute-on-chronic management	Haemoptysis and fungal disease complication management [9] Tools to distinguish exacerbations from baseline symptoms [3] Management strategies for PTLD with airflow obstruction [8] Acute complication management within a PTLD subtype-specific pathway [21]
Long-term monitoring and follow-up	Follow-up schedule: every 3 months in first year post-cure; 6-monthly evaluations up to 2 years post-treatment [3,21] Assessment battery: spirometry, chest radiography, 6MWT, QoL questionnaires, mental health assessment [3,7,21] Post-rehabilitation monitoring to confirm sustained benefits and detect deterioration [7]

Abbreviations: CT, computed tomography; EQ-5D, EuroQol five-dimension questionnaire; IMT, inspiratory muscle training; PFT, pulmonary function testing; PTLD, post-tuberculosis lung disease; PTLD-AFO, PTLD with airflow obstruction; QoL, quality of life; SGRQ, St George’s Respiratory Questionnaire; 6MWT, six-minute walk test.

### Global guidance documents

#### Search outcomes.

A total of 1,225 records were screened. Following screening, 51 documents were retrieved for full-text review, and 14 global guidance documents published between 2022 and 2025 were included in the review (Fig 1; S2 Table; Table 3). The included documents comprised two normative guidelines, six implementation resources, two policy briefs, two global strategic documents, one political declaration, and one monitoring report. The implementation resources included operational handbooks, strategic-planning guidance, a toolkit, and an information note. Nine documents were published by the World Health Organization (WHO), with the remainder originating from the United Nations General Assembly, the Stop TB Partnership, the Global Fund, and USAID. These documents cover political commitment, strategic direction, normative and policy guidance, operational support, and performance monitoring.

**Table 3:** Characteristics of included global guidance documents and orientating overview of intended use.

Guidance document (issuing body, year)	Document Type	Intended use / orientation
UN High-Level Meeting Political Declaration on TB (United Nations, 2023) [22]	Political declaration	High-level political commitments, priorities, and pledges intended to inform national and global TB agendas.
Global Plan to End TB 2023–2030 (Stop TB Partnership, 2022) [23]	Global strategic document	Global strategic plan outlining priority actions, coordination mechanisms, resource needs, and targets for TB elimination.
USAID Global Tuberculosis Strategy 2023–2030 (USAID, 2022) [13]	Global strategic document	Agency strategy outlining USAID’s priorities and approach across five pillars: Reach, Cure, Prevent, Innovate, and Sustain.
WHO Consolidated Guidelines for TB Treatment and Care (WHO, 2025) [14]	Normative guideline	Normative recommendations for TB treatment and care, corresponding to Module 4 of WHO’s consolidated TB guideline series
WHO Operational Handbook for TB Treatment and Care (WHO, 2025) [24]	Implementation resource	Operational companion to the WHO treatment and care guideline module, supporting implementation of WHO recommendations.
WHO Consolidated Guidelines for TB in Children and Adolescents (WHO, 2022) [25]	Normative guideline	Normative recommendations for TB management in children and adolescents, corresponding to module 5 of WHO’s consolidated TB guideline series.
WHO Operational Handbook for TB in Children and Adolescents (WHO, 2022) [26]	Implementation resource	Operational companion to the WHO guideline module for children and adolescents, supporting implementation of paediatric TB recommendations.
WHO Policy Brief on Tuberculosis-Associated Disability (WHO, 2023) [27]	Policy brief	WHO policy brief addressing TB-associated disability, including rehabilitation needs and programmatic actions.
Integrated Approach to Tuberculosis and Lung Health: Policy Brief (WHO, 2025) [28]	Policy Brief	Policy brief on integrating TB and lung health services; includes PTLD within integrated planning, outlines clinical/programme intersections, and presents a force-field analysis with strategic actions for service integration.
WHO Guidance for National Strategic Planning for Tuberculosis (WHO, 2022) [29]	Implementation resource	Operational guidance for developing, implementing, and evaluating national TB strategic plans aligned with End TB goals.
Implementing the End TB Strategy: The Essentials (WHO, 2022) [30]	Implementation resource	Implementation-oriented overview of core components and actions for End TB Strategy delivery.
Toolkit for Tuberculosis Program Essentials (Global Fund, 2023) [31]	Implementation resource	Toolkit outlining essential TB interventions and indicators for programme planning, funding, and monitoring.
Core Tuberculosis Information Note (Global Fund, 2022) [32]	Implementation resource	Information note providing guidance for Global Fund-aligned TB programming and funding applications.
Global Tuberculosis Report 2024 (WHO, 2024) [2]	Monitoring report	Annual global TB monitoring report summarising TB burden, service coverage, financing, and progress towards global TB targets.

#### Recognition of PTLD and related sequelae in global guidance documents.

Recognition of PTLD and related sequelae varied markedly across international TB guidelines (see Table 4). While some documents explicitly referenced PTLD and related sequelae, others omitted post-TB sequelae entirely or referred to them only indirectly. In many cases, these sequelae were mentioned only in umbrella terms such as “TB-associated disability,” with limited detail on specific manifestations or affected systems. Four documents contained no reference to post-TB sequelae whatsoever: WHO Consolidated Guidelines for TB Treatment and Care, WHO Global Tuberculosis Report 2024, USAID Global Tuberculosis Strategy 2023–2030, and the Global Fund’s Toolkit for Tuberculosis Program Essentials [2,13,14,31].

**Table 4:** Recognition of post-tuberculosis sequelae across global TB guidelines and policy documents.

Document	General recognition	Respiratory System Sequelae Recognised	Non-respiratory System Sequelae Recognised
UN High-Level Meeting Political Declaration on TB [22]	“Lifelong disabilities due to tuberculosis”	“Post-tuberculosis lung disease”	**X**
Stop TB Partnership Global Plan to End TB: 2023–2030 [23]	“Post-TB disease”	“PTLD”; “Post-TB lung disease”	Cardiovascular; neurological
USAID Global Tuberculosis Strategy 2023–2030 [13]	**X**	**X**	**X**
WHO Consolidated Guidelines for TB Treatment and Care [14]	**X**	**X**	**X**
WHO Operational handbook for TB Treatment and Care [24]	“Post-TB disability or sequelae”	“Post-TB lung disease (PTLD)”, “TB pulmonary sequelae”	Neurological; musculoskeletal; psychiatric
WHO Consolidated Guidelines for TB in Children and Adolescents [25]	“Post-TB sequelae”	**X**	Neurological – delineates specific diseases
WHO Operational Handbook for TB in Children and Adolescents [26]	“Post-TB health”	“Post-TB lung disease”	(Neurological, musculoskeletal, cardiopulmonary, endocrine) - delineates specific diseases
WHO Policy Brief on Tuberculosis-Associated Disability [27]	“TB-associated impairment”; “TB-associated disability”; “post-TB disease”	Respiratory - delineates specific diseases	(Psychiatric; neurological; renal; musculoskeletal) – delineates specific diseases
WHO Integrated Approach to Tuberculosis & Lung Health: Policy Brief [28]	“TB-associated disabilities”	“Post-TB lung disease” – delineates specific diseases	**X**
WHO Guidance for National Strategic Planning for Tuberculosis [29]	“TB-associated disability”	**X**	**X**
Implementing the End TB Strategy: The Essentials [30]	“TB-associated/related impairment and disability”	**X**	**X**
Global Fund Toolkit for Tuberculosis Program Essentials [31]	**X**	**X**	**X**
Global Fund Core Tuberculosis Information Note [32]	“Post-TB sequelae or disability”; “TB-associated disability”	“Post-TB lung sequelae”	**X**
WHO Global Tuberculosis Report 2024 [2]	**X**	**X**	**X**

Of the ten documents referring to post-TB sequelae, seven mentioned respiratory complications; the exceptions were the WHO Consolidated Guidelines for TB in Children and Adolescents, Implementing the End TB Strategy: The Essentials, and WHO Guidance for National Strategic Planning for Tuberculosis [25,29,30]. Five of these seven explicitly used the term “post-TB lung disease.” The exceptions were the WHO Policy Brief on Tuberculosis-Associated Disability and the Global Fund’s Core Tuberculosis Information Note, both of which described respiratory impairments without applying that label [27,32].

Non-respiratory sequelae were recognised in five of the ten documents. Three documents listed respiratory sequelae but no non-respiratory manifestations: the WHO’s Integrated Approach to Tuberculosis and Lung Health: Policy Brief, the UN High-Level Meeting Political Declaration on TB, and the Global Fund’s Core Tuberculosis Information Note [22,28,32]. The WHO Consolidated Guidelines for TB in Children and Adolescents showed the reverse pattern, listing neurological complications, without reference to respiratory sequelae [25].

#### Synthesised clinical recommendations for PTLD management in global guidance documents.

All extracted clinical recommendations are synthesised in Table 5; the verbatim extracted text is provided in S5 Table. Recommendations from high-level strategic documents, such as the WHO Guidance for National Strategic Planning for TB and the UN Political Declaration on TB, were excluded from this table as they were directed at policymakers rather than providing actionable clinical guidance [22,29]. These recommendations included primary prevention measures such as TB preventive treatment, tobacco cessation and lifestyle interventions, prompt diagnosis, adverse-event monitoring, and mental health support. Screening and diagnosis recommendations included structured post-TB respiratory assessment and selected radiological and functional investigations. Chronic management recommendations included multidisciplinary care, rehabilitation referral, psychosocial and palliative support, and symptom-guided therapy. Long-term monitoring and follow-up recommendations included clinical, radiological, and functional review, in some cases up to 2 years post-treatment, with additional follow-up guidance for drug-resistant TB. Recommendations for acute-on-chronic management were more limited and focused on continued care during exacerbations and investigation of underlying infections and comorbidities.

**Table 5:** Synthesised PTLD management recommendations from global guidance documents.

Domain	Recommendation	Target Population
Primary Prevention	TB preventive treatment [28] Tobacco cessation and lifestyle interventions [23,28] Prompt diagnosis and adverse-event monitoring [27] Routine mental health screening and support [23]	All TB patients
Screening and Diagnosis	Structured respiratory assessment post-TB [23,26,28] End-of-treatment chest X-ray; drug-resistant TB chest X-ray at 6 and 12 months [24,26] Paediatric: symptom screen, chest X-ray, spirometry; CT if needed [26] Early screening for impairments and overlapping lung disease [27,32]	TB survivors
Chronic Management	Multidisciplinary care: rehabilitation, social and mental health support, palliative care (including end-of-life support) [23,26–28] Specialised or community rehabilitation referral (for example, pulmonary rehabilitation; airway-clearance techniques) [23,26,32] Symptom-guided therapy: bronchodilators, opioids, oxygen [24,26]	PTLD patients
Acute-on-Chronic Management	Provide continued care and follow-up for acute exacerbations; investigate underlying infections and co-morbidities [28]	PTLD patients
Long-term Monitoring and Follow-up	Clinical, radiological and functional follow-up up to 2 years [23,26–28] Drug-resistant TB: chest X-ray and bacteriology at 6 and 12 months; specialist referral [24] Counselling and psychosocial support post-treatment [24]	TB survivors

Abbreviations: PTLD, post-tuberculosis lung disease; TB, tuberculosis; CT, computed tomography.

### PTLD management coverage across research publications and global guidance documents

**Fig 2:**
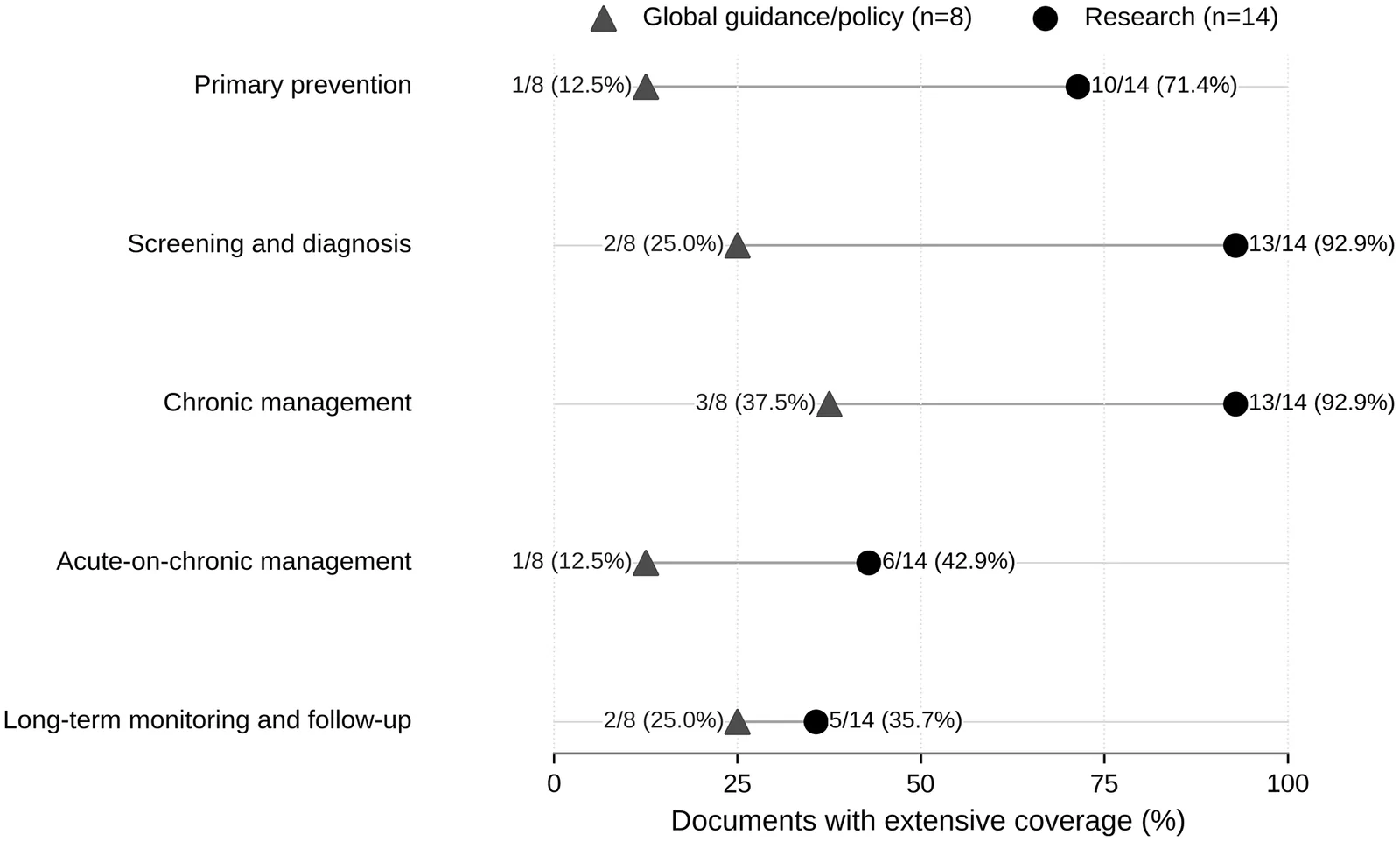
Extensive PTLD management coverage by evidence stream and management domain. The dot plot shows the proportion of included research publications (n = 14) and global guidance and policy documents with PTLD management recommendations (n = 8) that provided extensive coverage of each predefined PTLD management domain, as defined in the five-tier framework in the Methods. Full domain-by-document classifications, including lower coverage categories, are provided in S3 and S4 Tables. PTLD, post-tuberculosis lung disease.

Domain-level management coverage, classified using the five predefined PTLD management domains and five-tier coverage framework described in the Methods, differed between the research publications and global guidance documents (Fig 2; full domain-by-document classifications are provided in S3 and S4 Tables). In the research literature, screening and diagnosis and chronic management were the most consistently covered domains, each receiving ‘extensive coverage’ in 13 of 14 publications, followed by primary prevention in 10 of 14. Acute-on-chronic management and long-term monitoring and follow-up were less consistently covered, and four publications addressed all five domains with ‘extensive coverage’.

In the global guidance documents, 8 of 14 documents provided management recommendations relevant to PTLD. Among these eight documents, chronic management was the only domain addressed by all documents and had the highest number with ‘extensive coverage’, with three documents classified as such. Acute-on-chronic management had the lowest coverage. Primary prevention was also sparsely covered, with one document classified as having ‘extensive coverage’ and three as having ‘superficial coverage’, while screening and diagnosis and long-term monitoring and follow-up each received ‘extensive coverage’ in two documents. Only the WHO Integrated Approach to TB and Lung Health Policy Brief addressed all five domains with ‘extensive coverage’ [28].

## Discussion

This scoping review set out to provide a practical navigational map for the evolving and increasingly complex field of PTLD management. Many guidelines now acknowledge post-TB sequelae, but practical advice is often brief, ambiguous, or buried within broader frameworks, leaving implementers to bridge these gaps themselves.

Of the fourteen international guidelines and policy documents reviewed, only eight included any recommendations for PTLD. Major documents, including the WHO Consolidated Guidelines for TB Treatment and Care (2025), the Global Fund Toolkit, the WHO Global TB Report, and the USAID TB Strategy, do not reference post-TB sequelae at all. When sequelae are mentioned, such mentions are typically limited to generic terms such as “TB-associated disability”, with little actionable guidance. Most guidelines and research still focus largely on respiratory sequelae. Only five guidance documents address non-respiratory complications such as neurological, musculoskeletal, cardiovascular, or psychological consequences (see Table 4). This gap persists despite growing evidence that these may significantly contribute to the long-term burden of TB, especially in LMICs [3]. Equally concerning, given the inherent vulnerability of the paediatric population, are the WHO Consolidated Guidelines for Children and Adolescents [25]. Although this normative framework acknowledges neurological sequelae, it entirely omits respiratory consequences such as PTLD. Furthermore, it fails to provide any management recommendations even for the neurological conditions it does identify.

This fragmentation is further compounded by persistent gaps in the operationalisation of post-TB care. Although PTLD and other sequelae are increasingly acknowledged in the global health policy space, actionable guidance remains inconsistent and scattered. Policy commitments such as Sustainable Development Goal 3, while emphasising health equity and universal access, do not explicitly mention post-TB sequelae or disability. The WHO End TB Strategy envisions a world free of TB and suffering, which supports long-term care in principle, but post-TB outcomes are not integrated into key monitoring indicators or practical tools. This disconnect between research, policy, and programmatic action means that advances in understanding and recognition are not reliably translated into real-world implementation.

### Terminology and classification challenges

A recurring challenge across both research and guidance is inconsistent terminology and classification. Terms such as “rehabilitation” and “pulmonary rehabilitation,” and “disability” and “disease,” are used with differing scope, which complicates comparison across sources and may hinder programme design and implementation. In the research literature, pulmonary rehabilitation is generally described as a structured chronic-care intervention [7], whereas guidance documents often use broader rehabilitation and disability framings that encompass social, cognitive, and physical consequences beyond pulmonary function [26,27]. As one example, the WHO Policy Brief on Tuberculosis-Associated Disability acknowledges a range of impairments and respiratory manifestations but does not use the term PTLD, instead relying on broader labels such as “TB-associated disability,” “TB-associated impairment,” and “post-TB disease,” reflecting the continued lack of terminological specificity in global guidance [27]. This broader disability framing may support integration with rehabilitation and palliative care models, but it leaves the place of acute-on-chronic exacerbations less clearly specified within that framework [3,9,27]. Greater consistency in terminology and classification would improve comparability across studies and guidelines and help clarify the scope of post-TB care.

### Policy and practice gaps

Despite explicit policy commitments to TB elimination and universal health coverage, the practical integration of post-TB care remains limited. The Global Plan to End TB (2023–2030) situates TB and its sequelae within the framework of Universal Health Coverage, listing promotive, preventive, curative, rehabilitative, and palliative services among its aims. However, it does not specify operational approaches for the identification or management of PTLD or other sequelae. Similarly, USAID’s Global TB Strategy and the WHO End TB Strategy advocate for multisectoral, person-centred care, but provide minimal guidance on how to implement post-TB or PTLD-related interventions in practice [13,30]. As detailed in our results, these frameworks generally lack practical tools, specific recommendations, or indicators to support PTLD integration within routine TB care.

The depth of management content also differed markedly between the research literature and global guidance documents. All but one of the included research publications, which focused specifically on primary prevention [11], provided in-depth discussions of chronic management, and many proposed detailed management recommendations across the entire care cascade [3,4,9,15,21]. By contrast, where guidance documents addressed PTLD management at all, coverage was more often superficial and fragmented across care domains (Fig 2; S3 and S4 Tables). Part of this contrast follows from document type: broad political and strategic instruments, including the UN Political Declaration on TB and the global strategic plans included in this review, are designed to articulate high-level commitments rather than condition-specific clinical detail (Table 3). The same expectation does not extend to documents whose stated purpose is operational. Of the six documents classified as implementation resources (Table 3), the two WHO operational handbooks provided PTLD-relevant recommendations reaching extensive coverage in at least one management domain [24,26] (Table 5; S4 Table), whereas the remaining four, which support national strategic planning, End TB Strategy delivery, and Global Fund programming (Table 3), reached at most superficial coverage in any domain [29–32] (S4 Table). One of these, the Global Fund’s Toolkit for Tuberculosis Program Essentials, contained no reference to post-TB sequelae at all (Table 4).

Detailed management recommendations from the research community, such as those of Nightingale et al. [3], have not been incorporated into formal TB guidance or major funding and implementation tools. Furthermore, no reviewed guidance document included PTLD or post-TB sequelae among its programmatic indicators or reporting frameworks, most notably the WHO Global TB Report. Without PTLD-specific indicators, it is difficult for national programmes to prioritise or measure post-TB care. This lack of specificity and operationalisation represents a missed opportunity for driving implementation forward.

Even within the limited recommendations provided by the global guidance documents, some practical elements recurred. These included multidisciplinary care incorporating rehabilitation, psychosocial and palliative support, structured post-TB respiratory assessment, and clinical, radiological, and functional follow-up, in some cases up to 2 years post-treatment [23,26–28]. These elements may offer pragmatic starting points for integrating post-TB care into existing TB, rehabilitation, and primary health care platforms. By contrast, guidance on acute-on-chronic exacerbations remained comparatively limited, highlighting a domain in which operational development is still particularly weak [28].

These shortfalls should be read against a rapidly changing baseline. At the time of the 2018 review by Van Kampen et al., few international guidance documents made any reference to TB sequelae [12]; the recognition observed across the documents included here therefore represents meaningful movement over a relatively short period. This mirrors a post-TB research literature that has expanded substantially in recent years [3,10], while formal incorporation of post-TB sequelae into programmatic guidance and monitoring has only begun to emerge [10]. The persistence of operational gaps may thus reflect, in part, the time required for a recent and still-expanding evidence base to be translated into normative guidance and monitoring frameworks, rather than a static failure of translation.

### Implementation feasibility and health equity

Evidence-based recommendations for the care of post-TB sequelae are emerging, yet their feasibility varies across settings that differ markedly in infrastructure, workforce capacity, and funding. Successful adoption therefore depends on local adaptation and careful priority-setting, supported by context-specific burden data, pilot implementation studies, and economic analyses [33]. A broad portfolio of resources is already available to guide this work. Operational handbooks from the WHO in conjunction with the Global Fund Toolkit can be used immediately for programme design, policy integration, and advocacy [24,26,27,31]. Additionally, the WHO TB-associated Disability brief advocates for leveraging the WHO Rehabilitation 2030 initiative and The United Nations Convention on the Rights of Persons with Disabilities [27,34,35].

In parallel, implementation science tools, such as the WHO People-Centred Framework for TB Programme Planning and the WHO Handbook for Guideline Development, provide structured approaches for identifying barriers and tailoring strategies to local contexts [33,36]. For instance, the use of Patient Pathway Analysis (PPA) within the WHO People-Centred Framework can identify drop-off points in the continuum of care after treatment completion [33], and Evidence-to-Decision (EtD) frameworks can be used to weigh research evidence against implementation realities such as equity and resource use [36].

Post-TB sequelae care can also be integrated into existing service platforms, notably those aligned with the WHO Operational Framework for Primary Health Care, which is grounded in universal health coverage, primary health care, and person-centred principles [37]. While comprehensive, stand-alone guidance for post-TB sequelae is still evolving, current evidence and tools are sufficient to justify immediate action. Adapting and applying existing frameworks, rather than waiting for new guidelines, can accelerate service expansion. Policymakers and advocates can further leverage the high-level recognition of post-tuberculosis care needs, by bodies such as the WHO and the Stop TB Partnership to secure funding, draft policy briefs, and scale interventions.

### Strengths and limitations

This review has several strengths. We applied a single, predefined set of management domains and coverage categories across both peer-reviewed secondary literature and global guidance documents, so that PTLD-related content in each evidence stream was assessed against consistent and transparent criteria, and we provide the full domain-by-document classifications in S3 and S4 Tables to support reproducibility. To our knowledge, this is also the first synthesis to map both evidence streams together in this way since the 2018 review by Van Kampen et al., allowing current recognition to be assessed against a defined baseline [12].

This review also has several limitations. We mapped PTLD and related sequelae, but did not systematically assess adjacent domains such as palliative care, rehabilitation, end-of-life care, or broader primary health care, or exhaustively review allied-field frameworks (e.g., rehabilitation sciences and chronic respiratory disease management), even though these featured in several guidance documents. Our search, current through 15 May 2025, did not include EMBASE or Web of Science. We excluded national or regional policies and secondary sources lacking explicit management guidance. These choices may have omitted more recent publications, emerging viewpoints not yet formalised into clinical recommendations, and context-specific strategies. While we mapped acknowledgement of respiratory and non-respiratory sequelae in the global guidance documents, our extraction and synthesis of management recommendations were confined to PTLD and did not extend to non-respiratory conditions. Methodological constraints include language bias (only English-language sources), the absence of patient, caregiver, or community health worker perspectives, no critical appraisal of methodological quality or risk of bias, and no stakeholder consultation. Finally, variation in the structure, terminology, intended audience, document type, and expected level of clinical detail across sources, together with inconsistent use of terms such as “PTLD,” “post-TB sequelae,” or “TB-associated disability,” limited direct comparability within and between the two evidence streams.

### Research priorities and future directions

Addressing these persistent gaps and operational challenges will require coordinated, multi-pronged efforts. Future research should prioritise the development of consensus definitions and classifications for post-TB sequelae, especially in non-respiratory and paediatric domains, as well as the expansion of performance indicators for regular integration into TB monitoring frameworks at national and global levels. Implementation science is also needed to evaluate models of integrated, survivor-centred care, including how best to adapt NCD, rehabilitation, and palliative frameworks for TB survivors. Further studies should provide contextually grounded comparisons and clarify how findings from different settings translate into practice. Greater harmonisation and ongoing research investment are essential for advancing equity and patient-centredness.

## Conclusion

This scoping review shows that guidance on PTLD and other TB-associated sequelae remains scattered and inconsistently integrated across research and policy contexts. It highlights where recognition of PTLD and related sequelae has translated into actionable guidance, and where operational detail remains limited. Nevertheless, a clear consensus is emerging that TB survivors warrant ongoing attention for their long-term health needs. Moving forward, establishing consistency in definitions, classification, and programmatic indicators is crucial to unifying research, guiding policy, and standardising practice. Aligning these foundational elements will streamline the design and evaluation of post-TB interventions. With greater awareness, collaboration, and targeted research, post-TB care can be integrated into TB and chronic disease frameworks, supporting universal health coverage and human rights for all survivors. Strengthening the evidence base will further support strategies to improve outcomes, reduce disability, and ensure ongoing care for TB survivors. This review provides a practical foundation for harmonisation and future standard setting as the field evolves.

## Supporting information

S1 Checklist PRISMA checklist. Completed PRISMA Extension for Scoping Reviews (PRISMA-ScR) checklist. The PRISMA-ScR checklist is reproduced and used under the Creative Commons Attribution License (CC BY 4.0).
(PDF)

S1 Appendix Additional methodological information including search strategy and data extraction definitions. (DOCX)

S1 Table Research screened, reasons for inclusion/exclusion. (PDF)

S2 Table Guidelines Retrieval List: Full texts screened, reasons for inclusion/exclusion. (PDF)

S3 Table Coverage of PTLD management domains in included research articles. (DOCX)

S4 Table Coverage of PTLD management domains in global guidance documents. (DOCX)

S5 Table Verbatim clinical recommendations extracted from global guidance documents. (DOCX)
