# Supplementary material for: Mapping the research, policy, and clinical management landscape of post-tuberculosis lung disease: A scoping review
Source: PLOS Glob Public Health. 2026 Aug 28;6(8):e0007150. doi: 10.1371/journal.pgph.0007150 (PMC13524253; doi:10.1371/journal.pgph.0007150)
Supplement: Supplementary file 2 [file pgph.0007150.s003.pdf]

PMID	Title	Decision	Argument
33401266	Post-Tuberculosis Lung Disease: Clinical Review of an Under-Recognised Global Challenge.	Include.	Clinical review on PTLD (a broad, key sequela) published in 2021; meets all inclusion criteria.
38296392	Post tuberculosis lung disease and tuberculosis sequelae: A narrative review.	Exclude	This narrative review by Cupido & Günther (2024) primarily synthesizes epidemiology, pathophysiology, and risk factors of PTLD, offering only broad aspirational remarks on management rather than actionable clinical or programmatic recommendations. It therefore meets the “epidemiological burden/pathophysiology” exclusion criterion.
37455331	Post-tuberculosis lung disease and chronic obstructive pulmonary disease.	Exclude	The review summarizes epidemiology and pathophysiology without providing concrete management recommendations, and thus falls under the exclusion of studies centered on burden or pathophysiology rather than management.
33661159	Central nervous system tuberculosis.	Exclude	PTLD is not a principal focus
38225822	Long term management of people with post-tuberculosis lung disease.	Include.	Review published in 2024 with an explicit focus on the long-term management of PTLD.
31383464	Tuberculous meningitis: Challenges in diagnosis and management.	Exclude	PTLD is not a principal focus
33631128	Improving lung health in low-income and middle-income countries: from challenges to solutions.	Include.	Review published in 2021 covering post-tuberculosis lung disease as a key CRD and discusses solutions for management.
32032752	MDR/XDR-TB management of patients and contacts: Challenges facing the new decade. The 2020 clinical update by the Global Tuberculosis Network.	Exclude	Targets the MDR/XDR subgroup—a narrow population—so it’s excluded under “focused exclusively on a narrow population subgroup.”
33087432	Tuberculous Meningitis: Pathogenesis, Immune Responses, Diagnostic Challenges, and the Potential of Biomarker-Based Approaches.	Exclude	PTLD is not a principal focus

33716195	Global Tuberculosis Report 2020 - Reflections on the Global TB burden, treatment and prevention efforts.	Exclude	High-level policy reflection where PTLD is only mentioned in passing; does not present practical management guidance.
37194818	Diagnosis and management of post-tuberculosis lung disease.	Exclude	Classified as an editorial rather than peer-reviewed secondary literature (reviews, consensus statements, or conference reports); editorials are excluded by the methods.
32114203	Post tuberculosis treatment infectious complications.	Exclude	This review's scope is limited to infectious complications (e.g., aspergillosis, bronchiectasis superinfection) and does not provide the broad, programmatic management guidance for the full spectrum of PTLD sought by this review.
37035971	Post-TB health and wellbeing.	Include.	Clinical statement (consensus/guidance) from 2023 providing evidence-based guidance on the management of post-TB conditions.
34623101	Neurologic Complications of Tuberculosis.	Exclude	PTLD is not a principal focus
35124102	History of prevention, diagnosis, treatment and rehabilitation of pulmonary sequelae of tuberculosis.	Exclude	This 2022 review focuses on the historical evolution of pulmonary TB sequelae rather than providing practical clinical or programmatic management recommendations for PTLD, and thus falls outside the scope of management-focused secondary literature.
37179681	Tuberculosis of the Spinal Cord.	Exclude	PTLD is not a principal focus
35455684	Mycobacterium tuberculosis and Pulmonary Rehabilitation: From Novel Pharmacotherapeutic Approaches to Management of Post-Tuberculosis Sequelae.	Include.	Review from 2022 on the management of post-TB sequelae, specifically pulmonary rehabilitation. Meets inclusion criteria.
37902135	Burden, clinical features, and outcomes of post-tuberculosis chronic obstructive lung diseases.	Include.	Review from 2024 on PTLD-AFO, a key sequela, with a stated focus on management strategies. Meets inclusion criteria.

34058739	Defining Outcomes of Tuberculosis (Treatment): From the Past to the Future.	Exclude	A 2021 review focusing on standardized TB outcome definitions. It underscores the importance of post-treatment follow-up and suggests broader outcome frameworks, but does not offer any explicit clinical or programmatic PTLD management guidance.
38966406	Perspectives from the 2nd International Post-Tuberculosis Symposium: mobilising advocacy and research for improved outcomes.	Include.	Peer-reviewed conference report (2024) covering management, prevention, policy, and advocacy for post-TB outcomes. Meets inclusion criteria.
31038649	Managing severe tuberculosis and its sequelae: from intensive care to surgery and rehabilitation.	Exclude	This review focuses specifically on the management of *severe* TB cases requiring intensive care or surgery. While it discusses rehabilitation in this context, its primary focus is on acute, life-saving interventions rather than the broad, programmatic management of chronic PTLD, which is the scope of our review.
29978252	Tuberculosis of the central nervous system in children.	Exclude	PTLD is not a principal focus / narrow population focus
30057796	International research and guidelines on post-tuberculosis chronic lung disorders: a systematic scoping review.	Include.	Systematic scoping review from 2018 directly assessing literature on guidelines and research for PTLD.
33126930	Evidence for pulmonary rehabilitation in chronic respiratory diseases in sub-Saharan Africa: a systematic review.	Exclude	Does not explicitly focus on PTLD or give targeted recommendations, and the regional scope further supports exclusion.
37547037	Tuberculosis in older adults: challenges and best practices in the Western Pacific Region.	Exclude	Narrow population and regional focus
28932979	Tuberculous Meningitis in Children and Adults: New Insights for an Ancient Foe.	Exclude	PTLD is not a principal focus / narrow population focus
35811083	Point-of-care ultrasound for tuberculosis management in Sub-Saharan Africa-a balanced SWOT analysis.	Exclude	Narrow intervention focus
35753836	Breathing Back Better! A State of the Art on the Benefits of Functional Evaluation and Rehabilitation of Post-Tuberculosis and Post-COVID Lungs.	Include.	Scoping review from 2022 that describes and discusses post-TB sequelae and pulmonary rehabilitation.

38198346	Brazilian Thoracic Association recommendations for the management of post-tuberculosis lung disease.	Exclude	It is a national (Brazil-only) guideline rather than a truly global or multisite consensus. It therefore fits “focused exclusively on a narrow population subgroup (or region)” and does not meet the global-guidance scope in your research publications arm.
31982628	Rehabilitation, optimized nutritional care, and boosting host internal milieu to improve long-term treatment outcomes in tuberculosis patients.	Include.	Review from 2020 on rehabilitation and nutritional care to improve post-TB outcomes.
32912387	Post-tuberculosis lung health: perspectives from the First International Symposium.	Include.	Peer-reviewed conference report (2020) discussing priorities, gaps, and patient perspectives on post-TB sequelae management.
39236033	Post-tuberculosis lung disease: Addressing the policy gap.	Exclude	This article is a perspective or commentary piece that advocates for the development of clinical standards. As per our methods, only secondary literature presenting established recommendations (e.g., reviews, consensus statements) were included, while advocacy pieces and editorials were excluded.
32951405	Functional Outcomes in Spinal Tuberculosis: A Review of the Literature.	Exclude	PTLD is not a principal focus
38605448	Interventions to prevent post-tuberculosis sequelae: a systematic review and meta-analysis.	Include.	Systematic review and meta-analysis from 2024 evaluating interventions to prevent post-TB sequelae.
31964490	Intracranial CNS Infections: A Literature Review and Radiology Case Studies.	Exclude	PTLD is not a principal focus
38006959	Post-tuberculosis lung disease and inflammatory role players: can we characterise the myriad inflammatory pathways involved to gain a better understanding?	Exclude	Not clinically relevant
36598144	The rare manifestations in tuberculous meningoencephalitis: a review of available literature.	Exclude	PTLD is not a principle focus
35301102	Blue Skies research is essential for ending the Tuberculosis pandemic and advancing a personalized medicine approach for holistic management of Respiratory Tract infections.	Exclude	Not clinically relevant

32316300	The Growing Importance of Tuberculosis Preventive Therapy and How Research and Innovation Can Enhance Its Implementation on the Ground.	Exclude	PTLD is not a principle focus
38196060	Chronic Pulmonary Aspergillosis as a Considerable Complication in Post-Tuberculosis Lung Disease.	Exclude	Narrow disease manifestation focus
29943617	Could an increase in vigilance for spinal tuberculosis at primary health care level, enable earlier diagnosis at district level in a tuberculosis endemic country?	Exclude	PTLD is not a principle focus
37638142	South African Thoracic Society position statement on the management of non-cystic fibrosis bronchiectasis in adults: 2023.	Exclude	PTLD is not a principle focus
28963161	Tuberculosis of the spine with severe angular kyphosis: mean 34-year post-operative follow-up shows that prevention is better than salvage.	Exclude	PTLD is not a principle focus
33868917	Total hip arthroplasty in tubercular arthritis of the hip - Surgical challenges and choice of implants.	Exclude	PTLD is not a principle focus
37501183	Using a theory-informed approach to guide the initial development of a post-tuberculosis care package in British Columbia, Canada.	Exclude	Development of a care package in British Columbia is programmatic and region-specific, fitting the “narrow population/regional focus” exclusion.
31007099	Total hip arthroplasty in the setting of tuberculosis infection of the hip: a systematic analysis of the current evidence.	Exclude	PTLD is not a principle focus
31369544	Assessing quality of life for multidrug-resistant and extensively drug-resistant tuberculosis patients.	Exclude	Not clinically relevant
35055986	Tuberculous Meningitis in Children: Reducing the Burden of Death and Disability.	Exclude	PTLD is not a principle focus
31753065	Should we consider a 'fourth 90' for tuberculosis?	Exclude	PTLD is not a principle focus
37291686	Pharmacogenetics as part of recommended precision medicine for tuberculosis treatment in African populations: Could it be a reality?	Exclude	Narrow population focus
30203999	Total knee arthroplasty in the face of a previous tuberculosis infection of the knee: what do we know in 2018?	Exclude	Narrow interventional focus
32501258	Preclinical models to optimize treatment of tuberculous meningitis - A systematic review.	Exclude	PTLD is not a principle focus
32818138	Treatment Outcomes in Adult Tuberculous Meningitis: A Systematic Review and Meta-analysis.	Exclude	PTLD is not a principle focus

34496845	Tuberculosis related disability: a systematic review and meta-analysis.	Exclude	This review's main objective is quantifying TB-related disability burden rather than outlining PTLD management strategies, and thus fits the "epidemiological burden" exclusion clause.
30309300	Community-acquired acute meningitis and encephalitis: a narrative review.	Exclude	PTLD is not a principle focus
29322623	Clinical practice guidelines in India: Quality appraisal and the use of evidence in their development.	Exclude	Narrow regional focus
30685590	Improving treatment outcomes for MDR-TB - Novel host-directed therapies and personalised medicine of the future.	Exclude	This is an opinion-driven MDR-TB treatment viewpoint, not a PTLD-focused secondary review with post-treatment management recommendations. It falls outside the defined inclusion criteria.
30212165	Health Policy Analysis: Applications of Extended Cost-Effectiveness Analysis Methodology in Disease Control Priorities, Third Edition.	Exclude	Not clinically relevant
38125799	The tuberculous pleural effusion.	Exclude	Narrow disease manifestation focus
35840824	Providing Neurocritical Care in Resource-Limited Settings: Challenges and Opportunities.	Exclude	PTLD is not a principle focus
30764817	Out of the silos: identifying cross-cutting features of health-related stigma to advance measurement and intervention.	Exclude	PTLD is not a principle focus
35280919	Nanostructures for drug delivery in respiratory diseases therapeutics: Revision of current trends and its comparative analysis.	Exclude	Narrow interventional focus
36423650	The path to healthy ageing in China: a Peking University-Lancet Commission.	Exclude	Narrow population focus
30212102	principle Infectious Diseases: Key Messages from Disease Control Priorities, Third Edition.	Exclude	PTLD is not a principle focus
34615577	Clinical standards for the assessment, management and rehabilitation of post-TB lung disease.	Include.	Consensus-based clinical standards published in 2021 with an explicit focus on the assessment, management and rehabilitation of PTLD. The article provides practical clinical recommendations and meets the inclusion criteria.
40127662	Post-tuberculosis lung disease: towards prevention, diagnosis, and care.	Include.	Clinical review published in 2025 with an explicit focus on the prevention, diagnosis and care of PTLD. The article provides practical management content and implementation considerations and meets the inclusion criteria.
