# Supplementary material for: Mapping the research, policy, and clinical management landscape of post-tuberculosis lung disease: A scoping review
Source: PLOS Glob Public Health. 2026 Aug 28;6(8):e0007150. doi: 10.1371/journal.pgph.0007150 (PMC13524253; doi:10.1371/journal.pgph.0007150)
Supplement: Supplementary file 3 [file pgph.0007150.s004.pdf]

Title	Publication Year	Author	Decision	Reason	Url
Convention on the Rights of Persons with Disabilities	2006	Nations, United	Exclude	Focus too broad	https://www.ohchr.org/en/hrbodies/crpd/pages/conventionrightspersonswithdisabilities.aspx
Joint WHO/ILO policy guidelines on improving health worker access to prevention, treatment and care services for HIV and TB	2010	World Health Organization; International Labour Office	Exclude	Focus too broad	https://iris.who.int/handle/10665/44467
WHO Guideline: nutritional care and support for patients with tuberculosis	2013	World Health Organization	Exclude	Narrow focus	https://iris.who.int/handle/10665/94836
An Official American Thoracic Society/European Respiratory Society Policy Statement: Enhancing Implementation, Use, and Delivery of Pulmonary Rehabilitation	2015	Rochester, Carolyn L., et al.	Exclude	Narrow regional focus	https://www.atsjournals.org/doi/10.1164/rccm.201510-1966ST
Rehabilitation 2030 Initiative	2017	Organization, World Health	Exclude	Not primarily TB related	https://www.who.int/initiatives/rehabilitation-2030
2018 UN High Level Meeting	2018	UN	Exclude	Outdated version	https://cdn.who.int/media/docs/default-source/documents/tuberculosis/political-declaration-un-general-assembly-tb-tuberculosis77cd7a27-7e8d-4fbb-9729-a5dbd505798f.pdf?sfvrsn=4f4090dc_1&download=true
Treatment of Drug-Resistant Tuberculosis. An Official ATS/CDC/ERS/IDSA Clinical Practice Guideline	2019	Nahid, et al.	Exclude	Narrow regional focus	https://www.atsjournals.org/doi/10.1164/rccm.201909-1874ST
World Health Organization - 2019 - Rehabilitation in health systems guide for action.pdf	2019	WHO	Exclude	Not primarily TB related	https://www.who.int/publications/i/item/9789241515986

People-centred framework for tuberculosis programme planning and prioritization: user guide	2019	World Health Organization	Exclude	Not primarily TB related	https://iris.who.int/handle/10665/329472
The Deadly Divide_TB Commitments vs TB Realities FINAL HLM Report.pdf	2020	Stop TB Partnership	Exclude	Wrong document type	https://stoptb.org/assets/documents/communities/The%20Deadly%20Divide_TB%20Commitments%20vs%20TB%20Realities%20FINAL%20HLM%20Report.pdf
Objectives of the 6th Summit	2020	Weil, Diana	Exclude	Wrong document type	https://www.who.int/docs/default-source/hq-tuberculosis/2020-end-tb-strategy-summit-report-.pdf
OneImpact CLM Conceptual and Implementation Framework FN.pdf	2021	Stop TB Partnership	Exclude	Not primarily TB related	https://stoptbpartnershiponeimpact.org/resources/Conceptual%20Framework/OneImpact%20CLM%20Conceptual%20and%20Implementation%20Framework%20FN.pdf
Defeating meningitis by 2030: a global road map	2021	Organization, World Health	Exclude	Not primarily TB related	https://www.who.int/initiatives/defeating-meningitis-by-2030
Situational assessment checklist to guide implementation of the global strategy for tuberculosis research and innovation | TB Knowledge Sharing	2021	TBKSP	Exclude	Wrong document type	https://tbksp.org/en/node/2534
Director-General’s report on the implementation of the Global Strategy for TB research and innovation	2021	Kasaeva, et al.	Exclude	Wrong document type	https://www.ingentaconnect.com/content/10.5588/ijtld.21.0421
Operational Handbook - Module 4: Tuberculosis care and support | TB Knowledge Sharing	2022	WHO	Exclude	Duplicate	https://tbksp.org/en/node/2390
Framework for Collaborative Action on Tuberculosis and Comorbidities	2022	WHO	Exclude	Focus too broad	WHO i-Library (9789240055056)

TB services for persons with disabilities	2022	WHO	Exclude	Narrow focus	https://www.who.int/news/item/03-12-2022-transformative-solutions-for-equitable-access-to-tb-services-for-persons-with-disabilities
American Thoracic Society International Conference 2022: Highlights in Pulmonary Rehabilitation	2022	ATS	Exclude	Narrow regional focus	https://www.thoracic.org/members/assemblies/assemblies/pr/quarterly-bite/ats-international-conference-2022-highlights-in-pr.php
WHO consolidated guidelines on tuberculosis. Module 4: treatment. Tuberculosis care and support.	2022	WHO	Exclude	Outdated version	
WHO operational handbook on tuberculosis. Module 4: treatment and care	2022	WHO	Exclude	Outdated version	https://www.who.int/publications/i/item/9789240065116
WHO consolidated guidelines on tuberculosis. Module 4: Treatment and care.	2022	WHO	Exclude	Outdated version	
USAID Priority Country TB Roadmaps, Fiscal Year 2022 | Global Health	2022	USAID	Exclude	Wrong document type	https://www.usaid.gov/global-health/health-areas/tuberculosis/resources/news-and-updates/global-accelerator-end-tb/tb-roadmaps-fy-2022
WHO - Guidance for national strategic planning for tuberculosis	2022	WHO	Include		https://www.who.int/publications/i/item/9789240052055
USAID'S GLOBAL TUBERCULOSIS (TB) Strategy 2023- 2030	2022	USAID	Include		USAID's Global Tuberculosis Strategy 2023–2030 (PDF)
Operational Handbook - Module 5 - Management of TB in children and adolescents	2022	WHO	Include		https://tbksp.org/en/node/2179

WHO Consolidated Guidelines on Tuberculosis. Module 5: Management of Tuberculosis in Children and Adolescents	2022		Include		https://www.who.int/publications/i/item/9789240046764
Implementing the End TB Strategy: The Essentials	2022	WHO	Include		https://cdn.who.int/media/docs/default-source/hq-tuberculosis/implementing-the-end-tb-strategy-the-essentials_-2022-update.pdf
Global Plan to End TB 2023 - 2030	2022	Stop TB Partnership	Include		https://www.stoptb.org/global-plan-to-end-tb/global-plan-to-end-tb-2023-2030
UN High-Level Meeting on TB 2023 | Stop TB Partnership	2023	UN	Exclude	Duplicate	https://www.stoptb.org/un-high-level-meeting-tb/un-high-level-meeting-tb-2023
Guidance on Engagement of Communities and Civil Society to End Tuberculosis	2023	WHO	Exclude	Focus too broad	WHO i-Library (9789240080294)
Tuberculosis (TB) in the United States	2023	CDCTB	Exclude	Narrow regional focus	https://www.cdc.gov/tb/default.htm
Global Health Center | CDC	2023	CDC	Exclude	Narrow regional focus	https://www.cdc.gov/globalhealth/index.html
Pulmonary Rehabilitation for Adults with Chronic Respiratory Disease: An Official American Thoracic Society Clinical Practice Guideline	2023	Rochester, Carolyn L., et al.	Exclude	Not primarily TB related	https://www.ncbi.nlm.nih.gov/pmc/articles/PMC10449064/
Package of interventions for rehabilitation Module 4 Cardiopulmonary conditions	2023	WHO	Exclude	Not primarily TB related	https://iris.who.int/bitstream/handle/10665/370505/9789240071162-eng.pdf?sequence=1

Global Tuberculosis Report 2023	2023	WHO	Exclude	Outdated version	https://www.who.int/teams/global-programme-on-tuberculosis-and-lung-health/tb-reports/global-tuberculosis-report-2023
Core Tuberculosis Information Note	2023	Global Fund	Include		https://www.theglobalfund.org/media/4762/core_tuberculosis_infonote_en.pdf
UN TB meeting WHO - Sept 2023 - Political declaration	2023	UN	Include		https://documents-dds-ny.un.org/doc/UNDOC/LTD/N23/272/22/PDF/N2327222.pdf
Policy brief on tuberculosis-associated disability	2023	Organization, World Health	Include		https://www.who.int/publications/i/item/9789240077799
Toolkit for TB Program Essentials	2023	Global Fund	Include		https://resources.theglobalfund.org/media/14352/cr_tb-program-essentials_toolkit_en.pdf
Clinical Guidelines	2024	CDC	Exclude	Narrow regional focus	https://www.cdc.gov/tb/hcp/clinical-guidance/index.html
Resource & CDC involvement in LMICs: Global TB Epidemic	2024	CDC	Exclude	Narrow regional focus	https://www.cdc.gov/global-hiv-tb/php/globaltb/index.html
Tuberculosis (TB)	2024	CDC	Exclude	Narrow regional focus	https://www.cdc.gov/tb/index.html
The Performance-based Monitoring and Evaluation Framework – TB DIAH	2024	TB DIAH	Exclude	Wrong document type	https://www.tbdiah.org/assessments/pbmef/
Resources - Tuberculosis | World Health Partners	2024	World Health Partners	Exclude	Wrong document type	https://www.worldhealthpartners.org/cgcresources.php

TB Care Cascade | World Health Partners	2024	World Health Partners	Exclude	Wrong document type	https://www.worldhealthpartners.org/tb-care-cascade.php?page=project
End Treatment Assessment & Pulmonary Rehabilitation	2024	World Health Partners	Exclude	Wrong document type	https://www.worldhealthpartners.org/resources/cgc/brochures/End%20Treatment%20Assessment%20brochure%20updated%2002%2006%2023.pdf
Global Tuberculosis Report 2024	2024	WHO	Include		https://www.who.int/publications/i/item/9789240078345
Integrated Approach to Tuberculosis and Lung Health: Policy Brief	2025	WHO	Include		https://www.who.int/publications/i/item/9789240078390
WHO consolidated guidelines on tuberculosis. Module 4: treatment and care	2025	WHO	Include		https://www.who.int/publications/i/item/9789240107243
WHO consolidated operational handbook on tuberculosis. Module 4: treatment and care	2025	WHO	Include		https://www.who.int/publications/i/item/9789240108141
