# Supplementary material for: Mapping the research, policy, and clinical management landscape of post-tuberculosis lung disease: A scoping review
Source: PLOS Glob Public Health. 2026 Aug 28;6(8):e0007150. doi: 10.1371/journal.pgph.0007150 (PMC13524253; doi:10.1371/journal.pgph.0007150)
Supplement: Supplementary file 4 [file pgph.0007150.s002.docx]

# S1 Appendix. Additional methodological information

*This appendix provides supplementary methodological detail to support the Methods section, including the charting framework, search documentation, and extended extraction notes used in the review.*

## Search strategy

**Search timing and update note.** The search framework below presents the PubMed concept structure used for the initial search of peer-reviewed publications in April 2024. The PubMed and Semantic Scholar searches were rerun, and the institutional repositories originally consulted were rescreened, on 15 May 2025. Sources remained eligible if they were published or last updated between June 2017 and May 2025.

**PubMed search strategy**

\# CONCEPT 1: Post-TB sequelae

(

(post-tubercul*[tiab] OR sequela*[tiab] OR disabilit*[tiab] OR PTLD[tiab])

AND tuberculosis[tiab]

)

AND

\# CONCEPT 2: Clinical guidance

(

Practice Guideline[pt]

OR guideline*[tiab]

OR clinical standard*[tiab]

OR expert opinion*[tiab]

OR management[tiab]

OR intervention*[tiab]

OR prevention[tiab]

OR treatment[tiab]

OR rehabilitation[tiab]

OR recovery[tiab]

OR follow-up[tiab]

)

**Date limits and update**

- Initial database date filter: 29 June 2017 to 2 April 2024.
- Updated database and repository searches: 15 May 2025.
- Eligibility window applied to included sources: June 2017 to May 2025.
- Language eligibility applied during screening: English

**Global guidance search and screening**
Global guidance documents were identified through targeted institutional and platform searches, together with citation tracking. Candidate documents were initially assessed from their titles, summaries, stated purposes, and overall scope. Where relevance was unclear, documents were searched in full text using the terms “post-TB”, “after TB”, “disability”, “rehabilitation”, “palliation”, “complication”, “sequelae”, “post-treatment”, “long-term”, “follow-up”, and “chronic”. Keyword matches were reviewed in their surrounding context. Documents were not included or excluded solely on the basis of a keyword match; final eligibility was determined from the issuing authority, document type, global TB scope, and relevance to the broader TB care continuum, including post-treatment care or long-term outcomes after TB.

## Data extraction

**Basic charting fields**

| Category | Operational detail charted |
| --- | --- |
| Document details | Title; author(s) or issuing body; year of publication or last update; document type. |
| Scope and orientation | Brief summary of aim, focus, or scope; geographic focus; target population. |
| Recognition of respiratory sequelae | Whether lung or respiratory sequelae were mentioned; specific PTLD-related terminology used; and named respiratory manifestations where provided. |
| Recognition of non-respiratory sequelae | Other physiological systems or conditions mentioned, extracted only for global guidance documents. |
| Psychosocial sequelae | Whether psychosocial effects were mentioned generally or with specific examples such as depression, anxiety, stigma, or social/economic impacts. |
| Management content | Which PTLD management domains were addressed and what practical content, tools, recommendations, or follow-up actions were described. |
| Terminology and definitions | How terms such as PTLD, post-TB sequelae, post-TB disability, rehabilitation, or disability were used or defined. |
| Emergent implementation considerations | Any cross-cutting implementation issues, gaps, or ambiguities relevant to synthesis. |

**Extended charting notes for recognition of post-TB sequelae**

| Field | Extended note |
| --- | --- |
| Respiratory sequelae | Chart whether respiratory consequences were explicitly named, only implied, or accompanied by specific PTLD terminology and manifestations such as airflow obstruction, bronchiectasis, fibrosis, haemoptysis, or chronic respiratory symptoms. |
| Non-respiratory sequelae | Chart the physiological systems or conditions named, for example neurological, musculoskeletal, cardiovascular, endocrine, psychiatric, auditory, or visual sequelae. |
| Psychosocial effects | Chart whether psychosocial impacts were mentioned in general terms or with specific examples such as anxiety, depression, stigma, relationship disruption, or economic consequences. |

**Extended charting notes for the five PTLD management domains**

**1. Primary prevention**. Strategies to mitigate lung injury before or during TB disease, prior to PTLD diagnosis. This included early TB detection, prompt treatment initiation, host-directed approaches, and risk-factor interventions such as smoking cessation or other upstream measures where explicitly linked to prevention of post-TB morbidity.

**2. Screening and diagnosis**. Approaches used to identify PTLD or those at risk, usually after TB treatment. This included screening protocols, baseline end-of-treatment assessments, essential investigations, and any diagnostic algorithms or flowcharts.

**3. Chronic management**. Ongoing interventions for established PTLD intended to improve function, prevent progression, and optimise quality of life. This included pulmonary rehabilitation, pharmacological or procedural approaches, education, counselling, vaccination, and related long-term supportive care.

**4. Acute-on-chronic management**. Management of acute exacerbations or complications superimposed on chronic PTLD, such as haemoptysis, fungal disease, or acute worsening of respiratory symptoms. This included acute-event assessment tools, complication pathways, and condition-specific therapeutic strategies where described.

**5. Long-term monitoring and follow-up**. Ongoing review after initial management to monitor progression, detect complications, and guide further care. This included periodic clinical review, post-rehabilitation follow-up, and proposed follow-up intervals where stated. No consensus chronological cut-off was imposed a priori.

**Coverage classification framework used during charting**

| Category | Decision rule |
| --- | --- |
| Extensive coverage | Assigned when a document included substantive, focused content on the domain, such as a dedicated section, figure, table, practical tool, protocol, algorithm, or detailed discussion across more than one part of the document. Any one such feature was sufficient. |
| Superficial coverage | Assigned when the topic was explicitly discussed but only briefly, without a dedicated section, figure, table, flowchart, or practical detail. |
| Brief mention | Assigned when the topic was explicitly named only in passing, with no elaboration, context, or actionable guidance. |
| Acknowledged implicitly | Assigned when the topic was not explicitly named but could reasonably be inferred from broader language. Any such inference had to be justified in the extraction notes. |
| Not addressed or unclearly referenced | Assigned when the topic was absent or too vague to interpret confidently. |

**Classification rule.** Only one category was assigned per domain per document. When classification uncertainty remained between two categories, the more conservative lower category was used and accompanied by a brief extraction note.
