# Supplementary material for: Mapping the research, policy, and clinical management landscape of post-tuberculosis lung disease: A scoping review
Source: PLOS Glob Public Health. 2026 Aug 28;6(8):e0007150. doi: 10.1371/journal.pgph.0007150 (PMC13524253; doi:10.1371/journal.pgph.0007150)
Supplement: Supplementary file 5 [file pgph.0007150.s005.docx]

**S3 Table. Coverage of PTLD management domains in included research articles.**

| **Paper (clinical focus)** | **Primary prevention** | **Screening & diagnosis** | **Chronic treatment** | **Acute‑on‑chronic management** | **Long-term monitoring and follow-up** |
| --- | --- | --- | --- | --- | --- |
| **Kampen (2018)** | Extensive coverage | Extensive coverage | Extensive coverage | Not addressed or unclearly referenced | Brief mention |
| **Allwood (2020)** | Extensive coverage | Extensive coverage | Extensive coverage | Extensive coverage | Extensive coverage |
| **Allwood (2021)** | Extensive coverage | Extensive coverage | Extensive coverage | Extensive coverage | Brief mention |
| **Meghji (2021)** | Extensive coverage | Extensive coverage | Extensive coverage | Brief mention | Brief mention |
| **Migliori (2021)** | Extensive coverage | Extensive coverage | Extensive coverage | Superficial coverage | Extensive coverage |
| **Meca (2022)** | Superficial coverage | Extensive coverage | Extensive coverage | Acknowledged implicitly | Superficial coverage |
| **Nightingale (2023)** | Extensive coverage | Extensive coverage | Extensive coverage | Extensive coverage | Extensive coverage |
| **Sehgal (2024)** | Not addressed/uncl. ref. | Extensive coverage | Extensive coverage | Extensive coverage | Not addressed/uncl. ref. |
| **Akkerman (2020)** | Not addressed/uncl. ref. | Extensive coverage | Extensive coverage | Not addressed/uncl. ref. | Superficial coverage |
| **Pontali (2022)** | Not addressed/uncl. ref. | Extensive coverage | Extensive coverage | Not addressed/uncl. ref. | Superficial coverage |
| **Seo (2024)** | Extensive coverage | Extensive coverage | Extensive coverage | Extensive coverage | Extensive coverage |
| **Allwood (2024)** | Extensive coverage | Extensive coverage | Extensive coverage | Extensive coverage | Extensive coverage |
| **Alene (2024)** | Extensive coverage | Not addressed or unclearly referenced | Not addressed/uncl. ref. | Not addressed/uncl. ref. | Not addressed/uncl. ref. |
| **Meghji (2025)** | Extensive coverage | Extensive coverage | Extensive coverage | Superficial coverage | Superficial coverage |
