# Supplementary material for: Mapping the research, policy, and clinical management landscape of post-tuberculosis lung disease: A scoping review
Source: PLOS Glob Public Health. 2026 Aug 28;6(8):e0007150. doi: 10.1371/journal.pgph.0007150 (PMC13524253; doi:10.1371/journal.pgph.0007150)
Supplement: Supplementary file 6 [file pgph.0007150.s006.docx]

## S4 Table. Coverage of PTLD management domains in global guidance documents.

| **Guideline Document** | **Primary Prevention** | **Screening & Diagnosis** | **Chronic Management** | **Acute-on-Chronic Management** | **Long-term monitoring and follow-up** |
| --- | --- | --- | --- | --- | --- |
| **WHO Op. Handbook: Treatment & Care (2025)** | Acknowledged implicitly | Superficial coverage | Extensive coverage | Not Addressed | Extensive coverage |
| **WHO consolidated guidelines: treatment & care (2025)** | Not addressed | Not addressed | Not addressed | Not addressed | Not addressed |
| **WHO Op. Handbook: Children & Adolescents (2022)** | Acknowledged implicitly | Extensive coverage | Superficial Coverage | Superficial coverage | Superficial coverage |
| **WHO Consolidated Guidelines: Children & Adolescents (2022)** | Not Addressed | Not addressed | Not addressed | Not Addressed | Not addressed |
| **WHO Policy Brief: TB-Associated Disability (2023)** | Superficial coverage | Superficial coverage | Extensive coverage | Brief mention | Superficial coverage |
| **WHO Guidance for National Strategic Planning (2022)** | Superficial coverage | Acknowledged implicitly | Superficial coverage | Not Addressed | Not Addressed |
| **Implementing the End TB Strategy: The Essentials (WHO, 2022)** | Not Addressed | Not addressed | Not addressed | Not Addressed | Not Addressed |
| **WHO/Global TB Programme: Integrated Lung Health Brief (2025)** | Extensive Coverage | Extensive Coverage | Extensive Coverage | Extensive Coverage | Extensive Coverage |
| **Global Plan to End TB 2023 - 2030 (Stop TB Partnership, UNOPS, 2022)** | Superficial coverage | Superficial coverage | Superficial Coverage | Not Addressed or unclearly referenced | Superficial coverage |
| **UN Political Declaration on TB (2023)** | Acknowledged implicitly | Brief mention | Superficial coverage | Not Addressed | Brief Mention |
| **Global Fund Toolkit for TB Program Essentials (2023)** | Not Addressed | Not Addressed | Not addressed | Not Addressed | Not Addressed |
| **Global fund Core TB InfoNote** | Brief mention | Superficial coverage | Superficial coverage | Not addressed | Not addressed |
| **USAID Global TB Strategy (2023)** | Not Addressed | Not Addressed | Not Addressed | Not Addressed | Not Addressed |
| **WHO Global TB report** | Not addressed | Not addressed | Not addressed | Not addressed | Not addressed |
