# Supplementary material for: Mapping the research, policy, and clinical management landscape of post-tuberculosis lung disease: A scoping review
Source: PLOS Glob Public Health. 2026 Aug 28;6(8):e0007150. doi: 10.1371/journal.pgph.0007150 (PMC13524253; doi:10.1371/journal.pgph.0007150)
Supplement: Supplementary file 7 [file pgph.0007150.s007.docx]

S5 Table. Verbatim Clinical Recommendations Extracted from Global Guidance Documents

| Guideline Source | Management Domain | Extracted Recommendation (Verbatim Quote) | Target Population |
| --- | --- | --- | --- |
| WHO Integrated Lung Health Policy Brief (2025) | 1. Primary prevention | "Provision of TB preventive treatment" | General / Unspecified |
| WHO Integrated Lung Health Policy Brief (2025) | 1. Primary prevention | "Tobacco cessation programmes" | General / Unspecified |
| WHO Integrated Lung Health Policy Brief (2025) | 1. Primary prevention | "Other lifestyle interventions for prevention and comprehensive management of lung disease" | General / Unspecified |
| WHO Integrated Lung Health Policy Brief (2025) | 1. Primary prevention | "Recognize the synergistic impact of smoking on TB progression and lung cancer risk." | Patients with TB and smoking history |
| WHO Integrated Lung Health Policy Brief (2025) | 2. Screening and diagnosis | "...integrating respiratory assessment..." [as part of comprehensive care strategies that extend beyond bacteriological cure] | TB survivors |
| WHO Integrated Lung Health Policy Brief (2025) | 2. Screening and diagnosis | "[Consider as part of differential diagnoses] '...post-TB lung disease.'" | Individuals being evaluated for lung disease (e.g. with respiratory symptoms) |
| WHO Integrated Lung Health Policy Brief (2025) | 2. Screening and diagnosis | "Understand the challenges in diagnosing TB and lung cancer, because one condition can often obscure the clinical or radiological signs of the other." | Patients with clinical/radiological signs suggestive of TB or lung cancer |
| WHO Integrated Lung Health Policy Brief (2025) | 2. Screening and diagnosis | "...in cases where microbiological confirmation is not obtained, careful review of the clinical response over time is essential to detect potential treatment failure or misdiagnosis." | Patients treated for TB without microbiological confirmation |
| WHO TB Operational Handbook (2025) | 2. Screening and diagnosis | "it is prudent to undertake CXR at baseline, at the end of the second month of treatment and at the end of treatment, to document progress and to use for comparison if the patient’s clinical condition changes at any time after the achievement of treatment success (92)." | General / Unspecified (in context of pulmonary TB) |
| WHO TB Operational Handbook (2025) | 2. Screening and diagnosis | "Additionally, these patients should be thoroughly evaluated for post-tuberculosis lung disease (PTLD) following the completion of MDR/RR-TB treatment." | Patients with extensive pulmonary disease (MDR/RR-TB) |
| WHO TB Operational Handbook (2025) | 2. Screening and diagnosis | A checkmark (✓) is present for "Chest X-ray" under the "End of treatment" and "6 and 12 months post treatment" columns in the monitoring schedule table. | Patients on DR-TB treatment |
| WHO TB Operational Handbook (2025) | 3. Chronic management | "A chest radiograph at the end of treatment is also useful to optimally manage TB pulmonary sequelae after treatment (92)." | General / Unspecified (in context of pulmonary TB) |
| WHO TB Operational Handbook (2025) | 3. Chronic management | "Palliative care should be initiated immediately as needed and should be combined with TB treatment to relieve any suffering due to the disease, to the adverse effects of treatment, to comorbidities or to social problems." | People with TB |
| WHO TB Operational Handbook (2025) | 3. Chronic management | "Assessment and treatment of pain, dyspnoea and other physical symptoms due to TB." | People with TB |
| WHO TB Operational Handbook (2025) | 3. Chronic management | "Oxygen therapy may provide relief from mild dyspnoea and sometimes at least partial relief from moderate dyspnoea." | People with TB with mild to moderate dyspnoea |
| WHO TB Operational Handbook (2025) | 3. Chronic management | "Strong opioids such as morphine have been proven to relieve safely and effectively not only pain but also dyspnoea that is refractory to oxygen therapy and treatment of the underlying cause (106–112)." | People with TB with pain or refractory dyspnoea |
| WHO TB Operational Handbook (2025) | 3. Chronic management | "Is relief from dyspnoea offered? Oxygen may be used to alleviate shortness of breath in some cases... Bronchospasm can be controlled with a metered-dose inhaler with a spacer or mask. Morphine and other strong opioids provide effective relief from chronic or refractory dyspnoea..." | Patients with dyspnoea |
| WHO TB Operational Handbook (2025) | 3. Chronic management | "Is relief from pain and other symptoms offered? Paracetamol typically gives relief from mild pain. A strong opioid such as morphine should be used to treat moderate or severe pain..." | Patients with pain |
| WHO TB Operational Handbook (2025) | 5. Long-term monitoring and follow-up | "Counselling at the end of TB treatment is necessary to provide people with necessary information on the possibilities of post-TB symptoms or sometimes respiratory disability or sequelae; they need to access health care services for follow-up examinations when necessary." | People completing TB treatment |
| WHO TB Operational Handbook (2025) | 5. Long-term monitoring and follow-up | "It is important to continue to monitor the health of TB patients even after they have finished their medications for TB disease. They may need to be followed by a lung or heart doctor to help treat ongoing symptoms." | TB patients after treatment completion |
| Global Plan to End TB (2023-2030) | Primary Prevention | "Provide mental health evaluation, care and support during and post-treatment." | People with TB |
| Global Plan to End TB (2023-2030) | Primary Prevention | "Integrate TB care and support into other relevant health and nutrition programmes, with a focus on underlying conditions such as... tobacco cessation..." | People with TB |
| Global Plan to End TB (2023-2030) | Primary Prevention | "people with TB should receive mental health screening as a routine and early part of care, using established and validated screening methods, and be provided with appropriate support." | People with TB |
| Global Plan to End TB (2023-2030) | Diagnosis / Assessment | "assessing people with TB for PTLD when they reach the end of TB treatment" | People with TB at the end of treatment |
| Global Plan to End TB (2023-2030) | Rehabilitation | "identifying people with PTLD who should receive pulmonary rehabilitation (PR)" | People with PTLD |
| Global Plan to End TB (2023-2030) | Rehabilitation | "tailoring a PR programme to meet the needs of people and local communities" | People with PTLD and communities |
| Global Plan to End TB (2023-2030) | Long-term Monitoring & Follow-up | "Post-TB treatment follow-up for TB disease every six months up to two years" | General / Unspecified |
| WHO Policy Brief on TB-Associated Disability (2023) | Primary Prevention | "strengthening early access to quality diagnosis, treatment and care, including monitoring and management of adverse/side effects of anti-TB medicines" | "especially for persons with existing co-morbidities or under treatment for drug-resistant tuberculosis" |
| WHO Policy Brief on TB-Associated Disability (2023) | Screening & Diagnosis; Long-term Monitoring & Follow-up | "early identification and screening for impairments, disabilities, and subsequent rehabilitation needs, including follow-up and case management of at-risk populations during and after TB treatment;" | "at-risk populations" |
| WHO Policy Brief on TB-Associated Disability (2023) | Chronic Management | "The management of TB-associated impairments requires a holistic approach delivered in a timely fashion by a multidisciplinary healthcare team, that includes preventive and rehabilitation services aimed at improving the health and social outcomes of TB-affected people." | "TB-affected people" |
| WHO Policy Brief on TB-Associated Disability (2023) | Chronic Management | "provision of coordinated, multi-disciplinary care that includes rehabilitation, mental health care or other necessary healthcare for people with TB-associated disabilities, especially those with complex needs;" | "people with TB-associated disabilities, especially those with complex needs;" |
| Global Fund Core TB InfoNote (2023) | 2. Screening and diagnosis | "comprehensive assessment of patients... during and after TB treatment including care for post-TB sequelae and disability" | People with TB |
| Global Fund Core TB InfoNote (2023) | 2. Screening and diagnosis | "develop systems to facilitate timely investigation and diagnosis of conditions with overlapping chronic respiratory symptoms to facilitate appropriate care." | General / Unspecified |
| Global Fund Core TB InfoNote (2023) | 2. Screening and diagnosis | "Develop linkages to investigate and treat conditions other than TB with overlapping chronic respiratory symptoms or those that may be detected during CXR screening." | General / Unspecified |
| Global Fund Core TB InfoNote (2023) | 3. Chronic management | "...integrating care or supporting linkages to appropriate care during and after TB treatment including care for post-TB sequelae and disability, palliative care to alleviate suffering of patients, mental health and other chronic conditions like diabetes." | People with TB in the country |
| Global Fund Core TB InfoNote (2023) | 3. Chronic management | "Support community-based and led interventions and outreach services for... rehabilitation." | General / Unspecified |
| WHO Operational Handbook on TB - Children & Adolescents (2022) | Screening and Diagnosis | "Assessment at the end of TB treatment aims to identify children with post-TB lung disease and should be considered in children with more severe forms of PTB and in children who remain symptomatic at the end of TB treatment." | Children with more severe forms of PTB; children who remain symptomatic at end of treatment. |
| WHO Operational Handbook on TB - Children & Adolescents (2022) | Screening and Diagnosis | "Assessment should include a symptom screen, basic clinical examination and nutritional assessment." | Children with post-TB lung disease. |
| WHO Operational Handbook on TB - Children & Adolescents (2022) | Screening and Diagnosis | "Radiological imaging should be considered at the end of treatment to assess residual abnormalities, especially in children with more extensive disease..." | "Children, especially those with more extensive disease." |
| WHO Operational Handbook on TB - Children & Adolescents (2022) | Screening and Diagnosis | "Chest computed tomography (CT) scans...should be considered if there are substantial chronic or recurrent respiratory symptoms or signs and radiological abnormalities to evaluate the extent of post-TB lung disease..." | Children with substantial chronic or recurrent respiratory symptoms or signs and radiological abnormalities. |
| WHO Operational Handbook on TB - Children & Adolescents (2022) | Screening and Diagnosis | "Lung function testing should be considered in all children old enough to complete testing (typically aged 4 years and over) who had severe PTB and should include pre- and post-bronchodilation spirometry..." | Children (typically aged 4 years and over) who had severe PTB. |
| WHO Operational Handbook on TB - Children & Adolescents (2022) | Screening and Diagnosis | "[For Non-severe PTB] Clinical assessment and symptom/sign screen: Yes. If there are any residual symptoms, further investigations should be performed." | Children with non-severe PTB. |
| WHO Operational Handbook on TB - Children & Adolescents (2022) | Screening and Diagnosis | "[For Severe PTB] Imaging (CXR): Yes" | Children with severe PTB. |
| WHO Operational Handbook on TB - Children & Adolescents (2022) | Screening and Diagnosis | "[For Severe PTB] Lung function (spirometry): Yes" | Children with severe PTB. |
| WHO Operational Handbook on TB - Children & Adolescents (2022) | Screening and Diagnosis | "[For Severe PTB] Health-related quality of life: Yes" | Children with severe PTB. |
| WHO Operational Handbook on TB - Children & Adolescents (2022) | Chronic Management | "Medical treatment and long-term follow-up of children and adolescents with post-TB lung disease should be guided by symptoms, type of respiratory disease and additional investigations." | Children and adolescents with post-TB lung disease. |
| WHO Operational Handbook on TB - Children & Adolescents (2022) | Chronic Management | "Bronchodilation may be effective in children with responsive obstructive airway disease, but evidence is limited." | Children with responsive obstructive airway disease. |
| WHO Operational Handbook on TB - Children & Adolescents (2022) | Chronic Management | "The roles of pulmonary rehabilitation and airway clearance techniques require further investigation, and their use should be guided by symptoms and recurrent infections." | General / Unspecified (implied for symptomatic children). |
| WHO Operational Handbook on TB - Children & Adolescents (2022) | Chronic Management | "[For Dyspnoea] Use of handheld fan directed at face. Oxygen supplementation. Oral, intravenous or subcutaneous opioids..." | Children and adolescents with TB in need of palliative care. |
| WHO Operational Handbook on TB - Children & Adolescents (2022) | Chronic Management | "[For Cough] Over-the-counter cough medicines (e.g. honey preparations). Nebulized saline... Chest physiotherapy. Opioids if needed..." | Children and adolescents with TB in need of palliative care. |
| WHO Operational Handbook on TB - Children & Adolescents (2022) | Acute-on-Chronic Management | "Referral should be made to a respiratory or pulmonology clinic if available for the management of bronchiectasis." | Children with post-TB bronchiectasis. |
| WHO Operational Handbook on TB - Children & Adolescents (2022) | Acute-on-Chronic Management | "The roles of pulmonary rehabilitation and airway clearance techniques require further investigation, and their use should be guided by symptoms and recurrent infections." | Children with recurrent infections. |
| WHO Operational Handbook on TB - Children & Adolescents (2022) | Long-Term Monitoring & Follow-up | "If resources permit, long-term follow-up care should be established to manage these children [with destructive parenchymal disease and those with untreated airway complications]." | Children with destructive parenchymal disease or untreated airway complications. |
